# Enhanced Adipogenicity of Bone Marrow Mesenchymal Stem Cells in Aplastic Anemia

**DOI:** 10.1155/2014/276862

**Published:** 2014-04-30

**Authors:** Naresh Kumar Tripathy, Saurabh Pratap Singh, Soniya Nityanand

**Affiliations:** Stem Cell Research Facility, Department of Hematology, Sanjay Gandhi Post Graduate Institute of Medical Sciences, Raebareli Road, Lucknow 226014, India

## Abstract

Fatty bone marrow (BM) and defective hematopoiesis are a pathologic hallmark of aplastic anemia (AA). We have investigated adipogenic and osteogenic potential of BM mesenchymal stem cells (BM-MSC) in 10 AA patients (08 males and 02 females) with median age of 37 years (range: 06 to 79 years) and in the same number of age and sex matched controls. It was observed that BM-MSC of AA patients had a morphology, phenotype, and osteogenic differentiation potential similar to control subjects but adipocytes differentiated from AA BM-MSC had a higher density and larger size of lipid droplets and they expressed significantly higher levels of adiponectin and FABP4 genes and proteins as compared to control BM-MSC (*P* < 0.01 for both). Thus our data shows that AA BM-MSC have enhanced adipogenicity, which may have an important implication in the pathogenesis of the disease.

## 1. Introduction

Aplastic anemia (AA) is a bone marrow (BM) failure syndrome characterized by a fatty BM and peripheral pancytopenia. Defects in hematopoietic stem cells (HSC) as well in the BM stroma have been implicated in the pathogenesis of AA but the exact cause of the disease is still obscure [1]. We have previously shown in AA an increased apoptosis of BM cells [2], IL-8 levels [3], and expression of interferon-*λ* and tumor necrosis factor-*α* in BM T-cells as well as their increased levels in BM plasma [4]. These studies point towards the role of immune mechanisms and bone marrow microenvironment in the pathogenesis of the disease. Mesenchymal stem cells (MSC) are the key stem cells of the BM microenvironment that give rise to different stromal cell types including adipocytes, osteoblasts, endothelial cells, and stromal fibroblasts and maintain hematopoietic homeostasis in the marrow by cell-cell contact and by producing various hematopoietic cytokines and growth factors [5, 6]. The AA BM-MSC have been shown to have an abnormal gene expression profile [7] and abnormal immunological properties [8, 9] indicating a BM-MSC dysfunction in AA. It is also recently reported that adipocytes present in the BM suppress HSC maturation and differentiation and an imbalance between adipogenic and osteogenic differentiation of MSC may substantially influence hematopoiesis [10–12]. Thus, in order to further explore the role of BM-MSC in the disease, we have evaluated their adipogenic and osteogenic differentiation potential in patients with AA.

## 2. Materials and Methods

### 2.1. Subjects and Culture and Phenotypic Characterization of BM-MSC

Ten AA patients [12], 08 males and 02 females with median age of 37 years (range: 6 to 79 years), and the same number of age and sex matched controls were recruited in the study. After informed consent, 5 mL of BM was aspirated from the posterior superior iliac crest of each subject and BM-MSC were isolated, cultured and phenotypically characterized as per the standard protocol established in the lab [13]. The cells of 3rd passage were used in the experiments.

### 2.2. Adipogenic Differentiation

BM-MSC in 3rd passage were treated with adipogenic medium consisting of DMEM medium (Invitrogen) containing 10% FBS (Hyclone), 500 mM IBMX, 1 mM dexamethasone, 10 mg/mL insulin, and 100 mM indomethacin (adipogenesis kit, Chemicon). After 18 days, the cells were fixed and stained with oil red O stain to visualize the fat droplets in the cells.

### 2.3. Osteogenic Differentiation

BM-MSC in 3rd passage were treated with osteogenic medium consisting of DMEM medium (Gibco-Invitrogen) containing 10% FBS (Hyclone), 1 mM dexamethasone, 10 mg/mL glyceraldehyde 3-phosphate, and 0.1 mM ascorbic acid (osteogenesis kit, Chemicon). After 21 days, the cells were fixed with 4% paraformaldehyde and stained with alizarin red stain to visualize mineralization.

### 2.4. Reverse-Transcription Polymerase Chain Reaction (RT-PCR)

Expression of adiponectin, fatty acid binding protein 4 (FABP4) and osteopontin was done by RT-PCR. Total RNA of BM-MSC of AA patients and controls was extracted using RNeasy Mini RNA isolation kit (Invitrogen). One *μ*g of total RNA was reverse transcribed into cDNA using random hexamers (Invitrogen). The gene primers (MWG Biotech, http://www.mwg-biotech.com/) used were as follows. Adiponectin: (forward) 5′-AAGGAGATCCAGGTCTTATTGG-3′ and (reverse) 5′ACCTTCAGCCCCGGGTAC-3′ (accession number: NM_004797.2); FABP4: (forward) 5′-CCTTTAAAAATACTGAGATTTCCTTCA-3′ and (reverse) 5′- GGACACCCCCATCTAAGGTT-3′ (accession number: NM_001442.2); osteopontin: (forward) 5′- GGATCCCCAGATGCTGTGGCCACATG-3′ and (reverse) 5′- CTCGAGTTAATTGACCTCAGAAGATGC-3′ (accession number: NM_001040058.1); and *β*-actin: (forward) 5′-GCTCGTCGTCGACAACGGCTC-3′ and (reverse) 5′- CAAACATGATCTGGGTCATCTTCTC-3′ (accession number: BC016045). The amplicons were resolved on 2% agarose gel (Sigma-Aldrich) and pictures were acquired using gel documentation system (Alpha Imager, http://www.alphainnotech.com/).

### 2.5. Western Blotting

The BM-MSC treated with induction medium or untreated cells were homogenized in lysis buffer [10 mM Tris-Cl (pH 7.5), 50 mM NaCl, and 1% Triton-X-100 containing phenylmethylsulfonyl fluoride (1 mM) and protease inhibitor cocktail] and centrifuged at 12,000 xg for 15 min at 4°C and the supernatant was estimated for protein content. 100 *μ*g protein of each sample was subjected to 6% SDS-PAGE and electrotransferred onto nitrocellulose membrane. The membranes were incubated with antibodies against adiponectin (Abcam), FABP4 (R&D Systems; http://www.rndsystems.com/), osteopontin (Abcam, http://www.abcam.com/), and *β*-actin (R&D Systems) followed by incubation with HRP-conjugated corresponding secondary antibodies. The signals were detected using an enhanced chemiluminescence detection system (Amersham Biosciences, http://www.gelifesciences.com/).

### 2.6. Statistical Analysis

The results were calculated as mean ± SD. The difference between control and aplastic anemia patients was evaluated by Student's* t*-test.

## 3. Results

### 3.1. Morphology and Phenotypes

**Figure 1:**
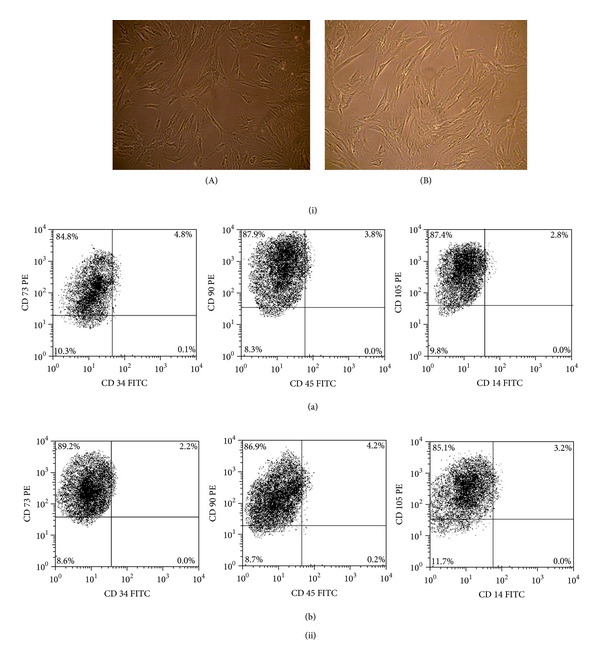
BM-MSC morphology and phenotype. (i) Fibroblastoid morphology of BM-MSC of (A) aplastic anemia patients and (B) control patients. (ii) Representative dot plots showing phenotype of BM-MSC of (a) aplastic anemia patients and (b) control patients.

The BM-MSC of patients with AA exhibited characteristic fibroblastoid morphology similar to those of controls. Flow cytometric analysis revealed that BM-MSC of AA patients and controls had similar expression of CD73 (96.77 ±2.03% versus 94.68 ± 2.26%), CD90 (97.96% ± 3.34% versus 98.86 ± 2.64%), and CD105 (92.28 ± 3.88% versus 89.28% ±  3.62%) (*P* > 0.5, for all) and absence of expression of CD34, CD45, and CD14 (Figure 1).

### 3.2. Adipogenic Differentiation and Expression of Adipogenic Transcripts and Proteins

**Figure 2:**
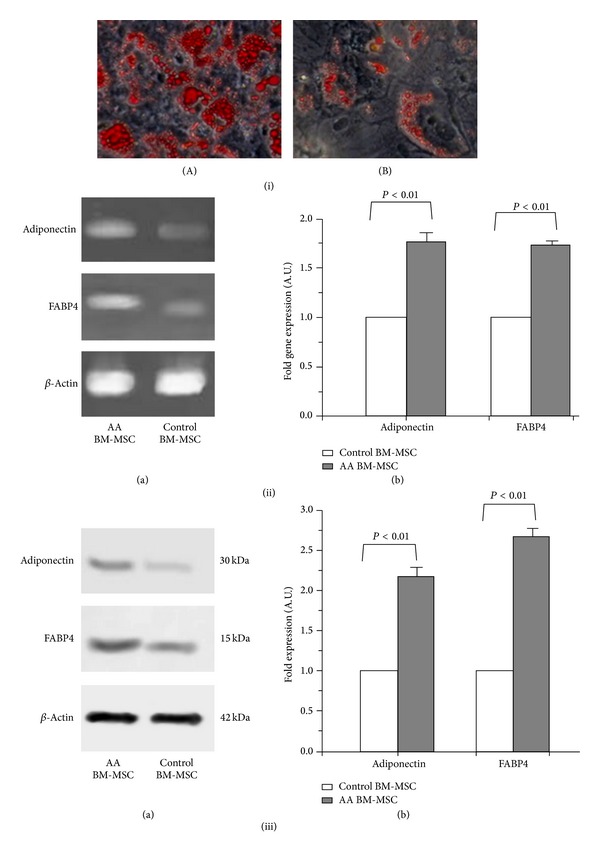
Adipogenic and osteogenic differentiation of BM-MSC of aplastic anemia patients. (i) Oil red O staining of adipocytes differentiated from BM-MSC of (A) aplastic anemia patients and (B) controls (bright field microscope view at 20x). (ii) Gene expression of adiponectin and FABP4 in adipocytes differentiated from BM-MSC of aplastic anemia patients and controls. (a) Representative gel pictures of RT-PCR. (b) Fold gene expression. (iii) Protein expression of adiponectin and FABP4 in adipocytes differentiated from BM-MSC of aplastic anemia patients and controls. (a) Representative Western-blot picture. (b) Fold expression of proteins.

Oil red O staining of the adipocytes differentiated from AA BM-MSC had a higher density and larger size of lipid droplets, as compared to controls (Figure 2: (iA) and (iB)). The RT-PCR and Western blot analysis showed significantly higher expression of Adiponectin and FABP4 transcripts and proteins, respectively, in the adipocytes derived from AA BM-MSC than those of controls (*P* < 0.01, for all) (Figure 2: (ii) and (iii)).

### 3.3. Osteogenic Differentiation and Expression of Osteopontin Gene and Protein

**Figure 3:**
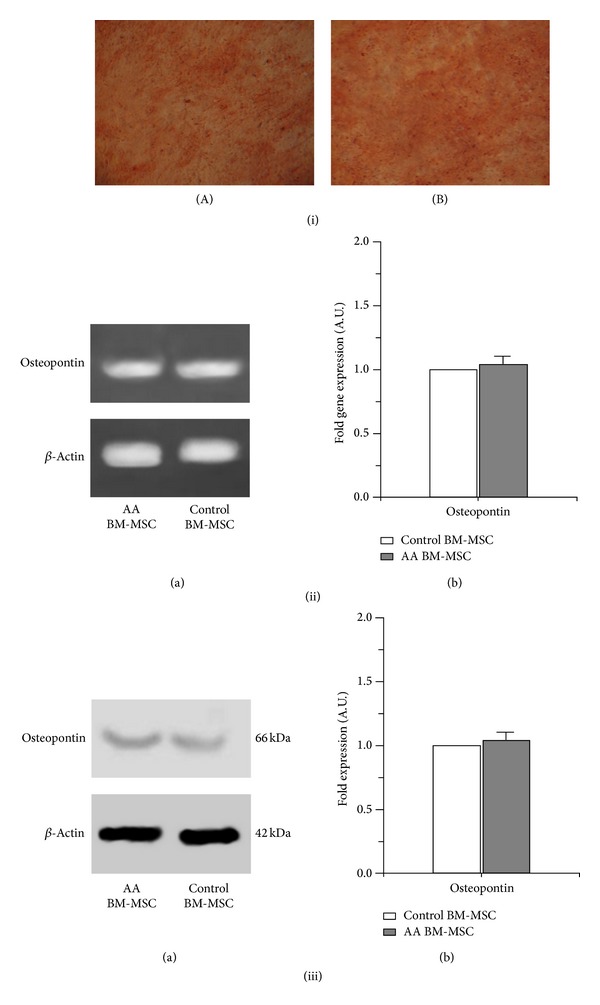
Osteogenic differentiation of BM-MSC of aplastic anemia patients. (i) Alizarin red staining of osteocytes differentiated from BM-MSC of (A) aplastic anemia patients (B) controls (bright field microscope view at 20x). (ii) Gene expression of osteopontin in osteocytes differentiated from BM-MSC of aplastic anemia patients and controls. (a) Representative gel pictures of RT-PCR. (b) Fold gene expression. (iii) Protein expression of osteopontin in osteocytes differentiated from BM-MSC of aplastic anemia patients and controls. (a) Representative Western-blot picture. (b) Fold expression of proteins.

The BM-MSC of patients with AA on treatment with osteogenic medium exhibited osteogenic differentiation similar to those of controls as shown by alizarin red staining (Figure 3: (iA) and (iB)). The RT-PCR and Western blot analysis showed no significant difference in the expression of osteopontin gene and protein, respectively, in osteocytes differentiated from BM-MSC of AA patients and those of controls (*P* > 0.5) (Figure 3: (ii) and (iii)).

## 4. Discussion

Our study shows that BM-MSC from patients with AA have morphology, phenotype, and osteogenic potential similar to those of controls but they exhibit an enhanced adipogenic potential as revealed by larger size and higher density of lipid droplets and a higher expression of adipogenic genes and proteins in adipocytes differentiated from AA patients as compared to controls.

The only one study available in the literature has demonstrated that AA BM-MSC have a normal phenotype and can be readily differentiated into adipocytes with increased expression of genes of adipocytokine signaling pathway including TRADD, PRKAB2, LEP, SLC2A1, and SOCS3 [7]. This study has also shown that AA BM-MSC are difficult to differentiate into osteoblasts but J. Li et al. have not studied the expression of the osteogenic genes or proteins in the differentiated cells. We have also observed a normal phenotype of BM-MSC from AA patients and demonstrated their enhanced adipogenicity by oil red staining of differentiated cells as well as by quantification of the adipogenic gene and proteins. However, we observed that expression of the osteopontin gene and proteins was similar to controls. Another study has reported that AA BM-MSC have a lower expression of GATA-2, which suppresses adipocytic differentiation, and a higher expression of peroxisome proliferator-activated receptor gamma that promotes adipocytic differentiation [14]. This study also lends support to our observation of enhanced adipogenic potential of AA BM-MSC.

Marrow adipocytes have long been viewed as space filler cells but some recent studies have shown that BM adipocytes are negative regulators of hematopoiesis and have a reciprocal relationship with osteoblasts that promote hematopoiesis [11, 14]. Moreover, it has also been reported that BM adipocytes produce neuropilin-1, adiponectin, and TNF-*α* and each of which has a suppressive effect on hematopoiesis [15]. We have shown that adipocytes differentiated from AA BM-MSC exhibit higher expression of adiponectin and FABP4 genes and proteins. Although the role of FABP4 in regulation of hematopoiesis is not known adiponectin is reported to potentially inhibit hematopoiesis. As BM adipocytes are a potent source of antihematopoietic cytokine TNF-*α* [15], the present study also supports our previous observation of increased levels of this cytokine in marrow plasma of patients with AA [4]. Thus the enhanced adipogenic potential of AA BM-MSC may contribute to the defective hematopoiesis in AA.

In summary, our study has demonstrated that BM-MSC of patients with AA possess an enhanced adipogenic potential which may have an important role in the pathogenesis of the disease. Further studies targeting molecular mechanisms involved in the hematopoietic inhibition by adipocytes and abnormalities in other biological properties of BM-MSC of AA patients would add new insights into the pathogenesis and treatment of the disease.
